# Evaluation of tongue squamous cell carcinoma resection margins using ex-vivo MR

**DOI:** 10.1007/s11548-017-1524-6

**Published:** 2017-01-27

**Authors:** Stefan C. A. Steens, Elise M. Bekers, Willem L. J. Weijs, Geert J. S. Litjens, Andor Veltien, Arie Maat, Guido B. van den Broek, Jeroen A. W. M. van der Laak, Jürgen J. Fütterer, Christina A. Hulsbergen van der Kaa, Matthias A. W. Merkx, Robert P. Takes

**Affiliations:** 1 Department of Radiology and Nuclear Medicine, Radboud University Medical Center, P.O. Box 9101, 6500 HB Nijmegen, The Netherlands; 2 Department of Pathology, Radboud University Medical Center, Nijmegen, The Netherlands; 3 Department of Oral and Maxillofacial Surgery, Radboud University Medical Center, Nijmegen, The Netherlands; 4 Department of Otorhinolaryngology and Head and Neck Surgery, Radboud University Medical Center, Nijmegen, The Netherlands

**Keywords:** Magnetic resonance imaging, Ex-vivo, Tongue, Squamous cell carcinoma, Validation

## Abstract

**Purpose:**

Purpose of this feasibility study was (1) to evaluate whether application of ex-vivo 7T MR of the resected tongue specimen containing squamous cell carcinoma may provide information on the resection margin status and (2) to evaluate the research and developmental issues that have to be solved for this technique to have the beneficial impact on clinical outcome that we expect: better oncologic and functional outcomes, better quality of life, and lower costs.

**Methods:**

We performed a non-blinded validation of ex-vivo 7T MR to detect the tongue squamous cell carcinoma and resection margin in 10 fresh tongue specimens using histopathology as gold standard.

**Results:**

In six of seven specimens with a histopathologically determined invasion depth of the tumor of ${\ge }3$ mm, the tumor could be recognized on MR, with a resection margin within a 2 mm range as compared to histopathology. In three specimens with an invasion depth of ${<}1$ mm, the tumor was not visible on MR. Technical limitations mainly included scan time, image resolution, and the fact that we used a less available small-bore 7T MR machine.

**Conclusion:**

Ex-vivo 7T probably will have a low negative predictive value but a high positive predictive value, meaning that in tumors thicker than a few millimeters we expect to be able to predict whether the resection margin is too small. A randomized controlled trial needs to be performed to show our hypothesis: better oncologic and functional outcomes, better quality of life, and lower costs.

## Introduction

Tongue squamous cell carcinoma (TSCC) is the most common malignancy in the head and neck region and is primarily treated surgically [1–5]. Goal of the surgical resection is to completely remove the tumor with adequate resection margins, to avoid the need for subsequent additional treatment such as repeated surgery or (chemo-) radiotherapy and to minimize functional impairment related to the procedure. Ultimately, optimizing the resection volume may result in better oncologic and functional outcomes, better quality of life, and lower costs [2].

The mucosal extension of the tumor usually can be evaluated by visual inspection. Although definite information on the deep resection margins requires postoperative histopathological analysis, peroperative information on the resection margins could help to optimize resection volume. Unfortunately, definite information on these resection margins is not yet available during surgery [1, 2]. Translation of information from preoperative imaging into the actual situation in the patient during surgery is challenging; macroscopic evaluation of a tumor in the tongue by palpation during surgery is difficult and inaccurate, and peroperative biopsies or frozen sections may suffer from sample error.

MR imaging using ultrahigh field strengths of 7T and above has recently been used to study healthy tissue ex-vivo such as lymph nodes [6] as well as neoplasms such as uveal melanoma [7], brain tumors [8, 9], carcinoma of the prostate [10–12], gastric and esophageal carcinoma [13–16] and liver metastasis [17]. One study on breast carcinoma patients using ex-vivo 1.5T MR [18] and one study on sarcoma patients [19] specifically aimed at evaluation of the resection margins, and one study using ex-vivo 1.5T MR evaluated the depth of invasion of TSCC [20]. To the best of our knowledge, this is the first study aiming at evaluating the feasibility and validity of ex-vivo 7T MR for the novel application of evaluation of resection margins in TSCC specimens. Furthermore, we summarize the future research and developmental improvements necessary for this technique to have the beneficial impact on clinical outcome that we expect: better oncologic and functional outcomes, better quality of life, and lower costs.

**Fig. 1:**
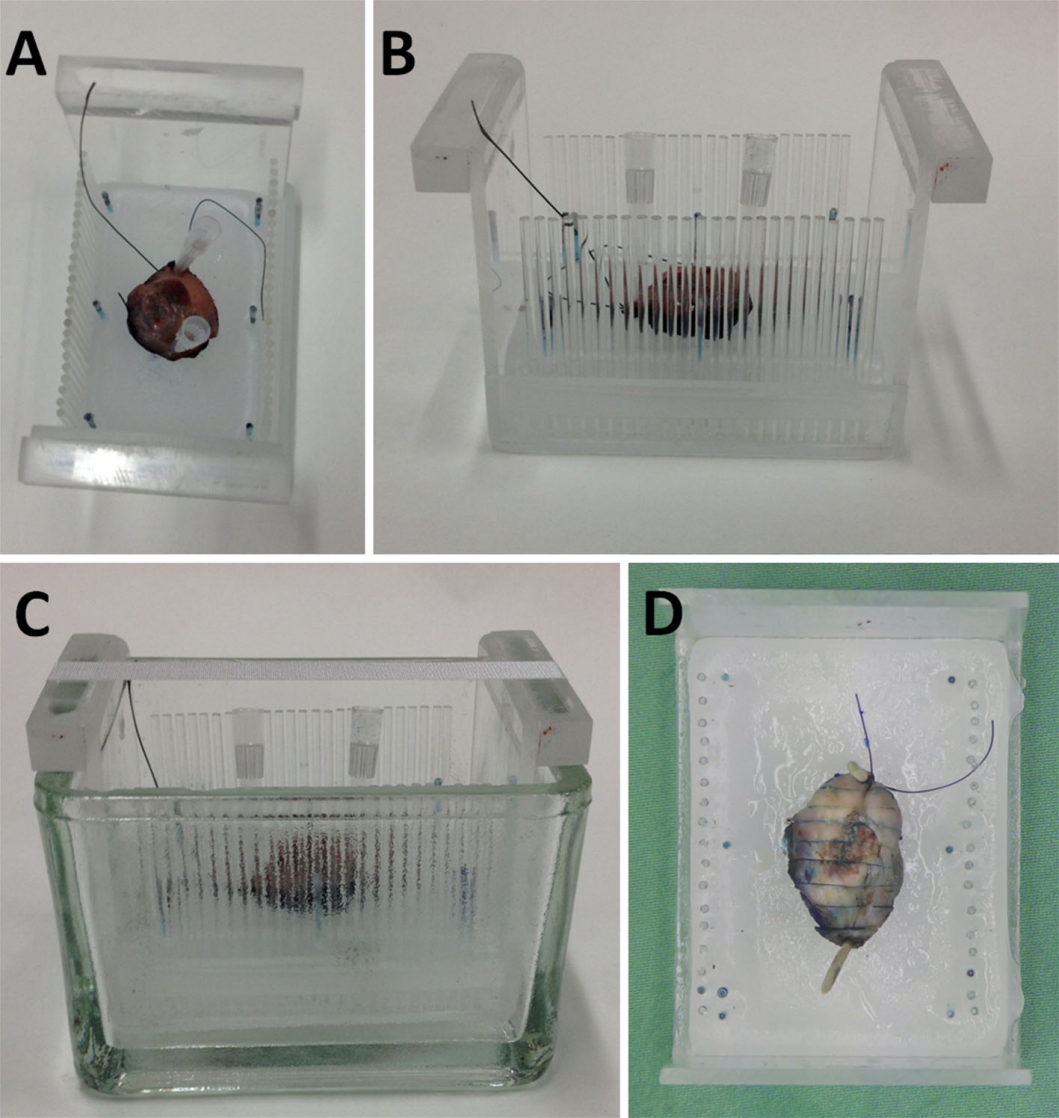
A specimen positioned on a bed of paraffin inside a specifically designed Perspex holder (**a**, from above; **b**, from aside), with pins on both sides 3 mm apart, then put into the glass container (**c**, from aside). In the middle, the holder contains water-filled tubes to facilitate matching between MR images and histopathology slices. The Perspex holder evolved during the study to this final configuration. After MR examination, the oil in the glass container was disposed off. After formalin fixation, the specimen was cut in 3-mm-thick slices from anterior to posterior using the pins in the holder and totally included (**d**, from above)

**Table 1:** MR sequence parameters

Sequence	Direction	Number of images	Slice thickness (mm)	Repetition time (ms)	Echo time (ms)	Averages	Matrix	Field of view (mm)	Scan time (ms)
T2-TSE	Sagittal	20–40	1	3080–6150	67	1	256 $\times$ 256	50 $\times$ 50–54 $\times$ 54	1$^{\prime }$51$^{\prime \prime }$–3$^{\prime }$42$^{\prime \prime }$
T2-TSE	Axial	25–52	1	3850–8610	13	1	256 $\times$ 256	50 $\times$ 50	2$^{\prime }$19$^{\prime \prime }$–5$^{\prime }$11$^{\prime \prime }$
T2-TSE	Axial	25–52	1	3850–8610	54	1	256 $\times$ 256	50 $\times$ 50	2$^{\prime }$19$^{\prime \prime }$–5$^{\prime }$11$^{\prime \prime }$
T2-TSE	Axial	9–20	3	2460–3070	54	1	256 $\times$ 256	50 $\times$ 50	1$^{\prime }$28$^{\prime \prime }$–1$^{\prime }$51$^{\prime \prime }$
DWI	Axial	48–56	1	2000	54	4	128 $\times$ 128	34$\times$34	6$^{\prime }$24$^{\prime \prime }$–7$^{\prime }$28$^{\prime \prime }$
DWI	Axial	48–56	1	2130	54	4	128 $\times$ 128	50 $\times$ 50	6$^{\prime }$49$^{\prime \prime }$–7$^{\prime }$57$^{\prime \prime }$

For number of images, repetition time, field of view and scan time ranges are given; variations between specimens were caused by large variations in volume of the specimens

*T2-TSE* turbo spin-echo T2-weighted sequence, *DWI* diffusion-weighted imaging

## Materials and methods

### Study population

This study was initiated within the framework of the Radboud University Medical Center MITeC initiative (Medical Innovation & Technology expert Centre) [21]. Between December 2013 and January 2015, ten patients with histologically proven TSCC of the lateral tongue were included prospectively, depending on anticipated immediate availability of the 7T MR machine after surgery (5 females, 5 males, age range 45–77 years). Patients received standard preoperative workup, surgery, and postoperative treatment when indicated. Validation of ex-vivo 7T MR data with histopathology was performed retrospectively after inclusion of the last patient.

### Preparation of the specimen before MR

Immediately after resection, the specimen was freshly transported at room temperature from the operating room to the Department of Pathology. Preparation consisted of inking and positioning the specimen with the largest resection plane downward on a bed of paraffin inside a customized Perspex holder that was optimized during the study with pins on both sides of the holder 3 mm apart (except for the first patient). The specimen then was fixed on the paraffin with wooden pins or pipettes and the complete setup was positioned in a glass container (Fig. 1).

### MR examination

After preparation at the Department of Pathology, the specimen was transported to the Preclinical Imaging Centre (PRIME) of the Radboud University Medical Center [22]. The glass container with the specimen was filled with a perfluoropolyether oil (Galden, Solvay Solexis, Thorofare, NJ, USA) to prevent susceptibility artefacts that would otherwise be caused by interfaces between tissue and air.

Ex-vivo MR was performed on a Bruker ClinScan horizontal-bore MR system, interfaced to a Siemens Syngo VB15 console (Bruker BioSpin, Ettlingen, Germany). MR images were recorded using an integrated circular polarized transmit/receive 1H volume coil with a free inner diameter of 154 mm. The scan protocol, which was optimized in one specimen not included in this study, included T2-weighted turbo spin-echo (T2-TSE) and one of two DWI HASTE sequences with *b*-values of 0-100-500-1000-1200 s/$\hbox {mm}^{2}$ (Table 1). Because of time restraints we did not include a 3D T1-weighted sequence. ADC maps were calculated using the standard postprocessing available in Siemens Syngo BV15. To facilitate matching of the MR images and histopathology slices, MR sequences were aligned to seven small water-filled tubes in the holder to mark the center of the specimen. The number of images was chosen to entirely cover the specimen front-to-back and repetition time and scan time were changed accordingly. The protocol parameters were set as to not exceed a total scan time of 1.5 h, including preparation and positioning of the MR sequences.

### Preparation of the specimen after MR

After the MR examination, the oil in the glass container was disposed off and the specimen inside the glass container was transported back to the Department of Pathology, formalin-fixed overnight, and cut in 3-mm-thick slices from anterior to posterior by using the pins in the holder 3 mm apart to conduct the knife (Fig. 1). Digital photography of the gross slices was performed. All slices were paraffin-embedded, processed and 4-$\upmu$m-thin tissue sections were cut and stained with hematoxylin and eosin. The histopathological diagnoses were classified according to the 2005 WHO criteria [23]. The maximal invasion depth of the tumor and minimal resection margin for the specimen were evaluated according to standard protocol.

**Table 2:** Patient characteristics and results from histopathology (PA) and ex-vivo 7T MR (MR)

cTNM	Invasion depth (mm)		Resection margin (mm)		Adjuvant treatment
	PA	MR	PA	MR	
cT2	5.0	5.5	4.0	4.7	None (follow-up)
cT2	0.6	–	11.0	–	None (follow-up)
cT1	8.0	7.7	0.6	1.1	RT (narrow resection margin, infiltrating growth pattern)
cT2	3.0	3.2	8.0	8.2	None (follow-up)
cT2	0.4	–	3.5	–	None (follow-up)
cT2	4.5	3.8	2.0	2.4	RT (infiltrating growth pattern)
cT2	3.0	3.6	4.0	4.1	RT (perineural extension)
cT2	7.0	7.1	4.0	3.8	RT (infiltrating growth pattern)
cT1	0.9	–	2.0	–	None (follow-up)
cT4a	7.0	6.2	2.0	3.8	None (follow-up)

*RT* postoperative radiotherapy

### Histopathological analysis

Histopathological examination was in no way hampered by the ex-vivo MR procedure as no tissue changes have been observed as compared to the routine procedure. Slices were scanned with the 3DHistech Panoramic250 (SYSMEX Belgium N.V., Hoeilaart, Belgium) and Olympus Dotslide (Olympus Nederland B.V., Zoeterwoude, Netherlands). A senior pathologist [CH] and a pathologist in training [EB] together annotated tumor borders and the associated inflammatory infiltrate on each 4-$\upmu$m-thin tissue section using Aperio ImageScope v11.2.0.780 (Aperio Technologies, Inc. Vista U.S.).

### MR analysis and correlation to histopathology

Using the photographs of the 3-mm gross slices and annotated 4-$\upmu$m-thin tissue sections, evaluation of the tumor on MR was performed in a non-blinded fashion by the pathologist in training [EB] together with a head and neck radiologist [SS]. Using integrated information from all MR sequences available, maximal invasion depth and minimal margin to the nearest resection plane were noted using a digital ruler.

## Results

In six of seven specimens that showed an invasion depth of the tumor of ${\ge }3$ mm at histopathology, the tumor could be recognized and delineated on the combination of MR sequences. In one of seven specimens with an invasion depth of the tumor of ${\ge }3$ mm at histopathology, the tumor was visible on MR, but delineation was difficult. In the three specimens with an invasion depth of the tumor of ${<}1$ mm, the tumor was not visible on MR. These specimens mainly contained dysplasia with only minor invasive carcinoma components. In the specimens where the tumor could be delineated, resection margin as measured on MR was within a 2 mm range as compared to histopathology (Table 2).

**Fig. 2:**
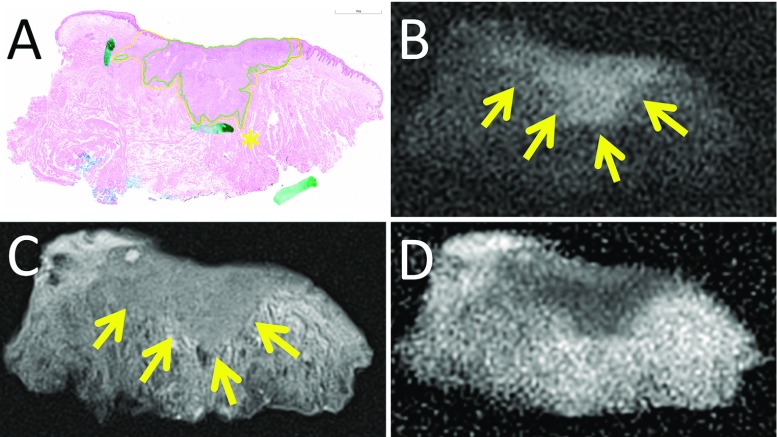
Example of MR images of a specimen containing TSCC, extending into the musculature. **a** $4\,\upmu$m hematoxylin and eosin stained histopathological section with tumor (*green*) and associated inflammatory infiltrate (*yellow*) annotated (*green marks* from standard clinical handling of the specimen), **b** DWI with *b*-value of 1000 s/$\hbox {m}^{2}$ and slice thickness of 1 mm, **c** T2-TSE with TE of 13 ms and slice thickness of 1 mm, **d** ADC map. The TSCC is marked with *arrows* on b and c. The slight difference in configuration of the specimen between MR and histopathology is caused by gravity in the Perspex holder in the MR machine. At all three MR images, the tumor can clearly be delineated. At this histopathological section, the minimal resection margin was measured (*asterisk* in **a**). However, the fissure at this location of minimal resection margin at the histopathological section was not visible at MR due to lower resolution, and minimal resection margin at MR would have been measured at a different section resulting in a slight difference. At the MR images, exact delineation of the mucosal resection plane is difficult

**Fig. 3:**
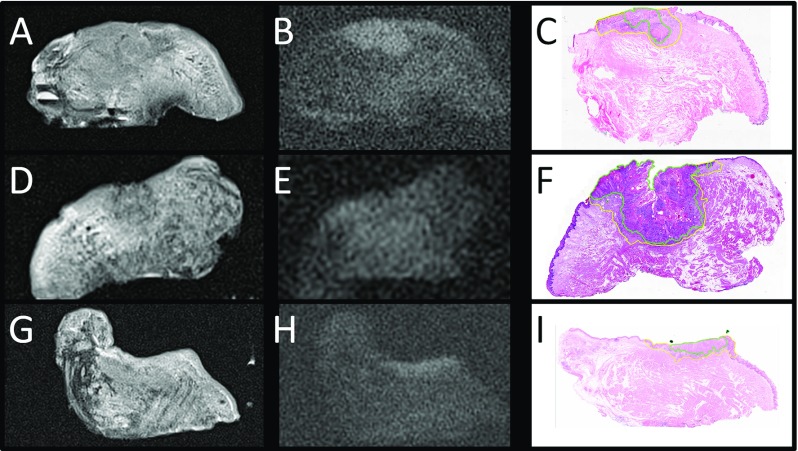
Example of MR images of three different specimens (**a**–**c**, **d**–**f** and **g**–**i**). **a**–**d**–**g** T2-TSE with TE of 13 ms and slice thickness of 1 mm, **b**–**e**–**h** DWI sequence with *b*-value of 1000 s/$\hbox {m}^{2}$ and slice thickness of 1 mm; **c**–**f**–**i** corresponding $4\,\upmu$m hematoxylin and eosin-stained histopathological section with tumor (*green*) and tumor-related infiltrate (*yellow*) annotated. The MR images show the differences in signal intensity changes in different specimens, with the tumor clearly visible in T2-TSE (**a**) and DWI (**b**) images in the first specimen, clearly visible on T2-TSE image (**d**) but less conspicuous on DWI image (**e**) in the second specimen, and more difficult to be recognized on T2-TSE (**g**) than on DWI (**h**) in the third specimen. The tumor seems to be separately recognizable from the associated inflammatory infiltrate in the first specimen, but the infiltrate is too small to be recognized on the MR images in the second and third specimen. As in the specimen in Fig. 2, exact delineation of the mucosal resection plane on MR is difficult

The lower resolution of MR as well as slight differences in configuration of the specimen as compared to histopathology caused different positioning of the measurements of invasion depth and resection margins (Fig. 2). Although the margins to the deep resection plane could be measured rather easily, exact identification of the mucosal resection plane on MR was more difficult in three out of seven specimens. We could not consistently recognize small bands of associated inflammatory infiltrate on MR as depicted on histopathology (Fig. 3). For individual sequences, signal intensity changes as compared to the surrounding tissues were not consistent.

Based on postoperative histopathological analysis and according to the protocols, six out of ten patients did not need additional treatment. In four out of ten patients, histopathological tumor characteristics such as a perineural or infiltrative growth pattern necessitated additional radiotherapy (Table 2).

## Discussion

To the best of our knowledge, this is the first study showing the feasibility and validity of ex-vivo 7T MR for the novel application of evaluation of resection margins in TSCC specimens. The results of our study suggest that ex-vivo 7T is expected to have a low negative predictive value, meaning that it will be difficult to a) detect very small tumors and b) predict that the resection margin is large enough given the inability to visualize microscopic invasive growth patterns. However, important is that we expect a high positive predictive value, meaning that in tumors thicker than a few millimeters we expect to be able to predict whether the resection margin is too small.

Overall, a good correlation existed between MR and histopathology for minimal resection margin and maximal depth of tumor invasion for TSCC specimens with an invasion depth of ${\ge }3$ mm. This is in accordance with the only previous ex-vivo study in TSCC specimens that we are aware of, measuring maximal depth of tumor invasion on a 1.5T MR [20]. Factors contributing to the observed differences in invasion depth and resection margins in the current study include the lower resolution of MR as compared to histopathology (resulting in more difficult delineation of the tumor) and slight differences in configuration of the specimen due to deformability and effect of gravity in the Perspex holder. Also, some shrinking of the specimen may have occurred between MR and the final histopathological examination [5]. These factors may hamper exact comparisons between ex-vivo MR images and histopathology slices, which is of utmost importance to prove that MR is accurate. We have tried to overcome the problem by using a home-made Perspex holder, aligning MR to water-filled tubes and using pins inside which exactly fit the pathologists’ knife. Alignment was not perfect and the technical setup has to be improved, either by physical restraints of the specimen, or by rigid and / or non-rigid registering of MR and histopathology data by postprocessing software. However, exact comparison between ex-vivo MR images and histopathology slices is important for validation of the technique only, as this will not be necessary in clinical practice.

Since our ultimate goal is to provide the surgeon with information on the resection margins during surgery, scan times must be kept as low as possible. On the other hand, resolution must be optimized to increase the detection of small, spiculated tumoral extensions as good as possible. In this pilot study, we chose to use the high signal-to-noise ratio of 7T MR as compared to systems with lower field strengths to acquire high-resolution images, at relatively low scan times [24]. At present, however, the total time for transportation, preparation and MR examination of the specimen was too long for clinical application. Given the positive results of this pilot study at a 7T MR system, we will now perform a subsequent study on a clinical 3T MR system, which is more widely available. In our institution, 3T MR is even located in the operating theater, thereby significantly reducing transportation time. One of the main issues to overcome the challenge to increase resolution and decrease scan time will be to focus on performing one optimized T2-TSE and one DWI sequence only.

We expect a clear advantage of the application of ex-vivo MR on clinical outcome for part of our patients. By providing information on the resection margins during surgery, ex-vivo MR may aid the surgeon in guiding peroperative biopsies or frozen sections, and optimizing the resection. As a result, the functional disability resulting from the resection may decrease, as well as the need for repeated surgery or postoperative (chemo-) radiotherapy, with a decrease in additional patient burden and costs to society. This may be true for many other (soft tissue) tumors where clearance of resection margins is of utmost importance and a one-step treatment can increase quality of life as compared to a treatment with repeated surgery or chemoradiotherapy. However, as stated above, the strength of the technique will lie in the positive predictive value predicting insufficient margins, and not in the negative predictive value predicting that the tumor margins are sufficient. In either case, histopathology will always be gold standard.

Disadvantages are mainly logistic. First, in those cases without neck dissection or sentinel lymph node biopsy performed during the MR examination, the patient will have to be kept under general anesthesia just waiting on the MR results. However, the vast majority of patients with TSCC will undergo a procedure of the neck as well during which MR can be performed. Second, an additional MR leads to additional costs, although it is expected that these additional costs will be largely outweighed by the savings from repeated surgeries and additional (chemo-) radiotherapy.

Apart from the technical issues that have to be solved, limitations were related to the explorative nature of this study. First, the sample size was small. Second, the configuration of the Perspex holder containing the specimen was not similar for all specimens. The holder containing the specimen was not anticipated but was developed during the study, as we experienced difficulties in visually matching MR and histopathological data in the first patients [17, 25, 26]. Third, our study was non-blinded. We chose to first prove the technical feasibility of ex-vivo 7T MR to visualize the tumor and learn to interpret the MR images, before evaluating the whole pipeline.

Apart from the next steps to decrease scan time, to increase resolution, and to implement the technique on a 3T machine, we have to optimize correlation of MR data with histopathology to understand signal intensity changes on specific sequences. For example, with increased resolution and optimized correlation with histopathology, we will investigate whether histological features such as hemangiosis, lymphangiosis or perineural invasion has any influence on the MR images. Unfortunately, the sample size of our current study was too small to recognize specific patterns on the specific sequences, and it was the combination of sequences that led to identification of the tumor and resection margins in most patients.

In all of these research and developmental innovations, as a team mainly composed of clinical doctors we actively seek collaborations with industrial partners and technical and IT experts. We will perform a randomized controlled trial to prove that the additional information on resection margins during surgery actually leads to better functional outcomes for the patient. Furthermore, we plan on making a direct comparison of ex-vivo MR and ex-vivo ultrasound. For unknown reasons peroperative intraoral and ex-vivo ultrasound has not gained wide application, although in the last decade some promising results with respect to assessment of the TSCC resection margin have been achieved [27–31].

In conclusion, in this study we have shown the feasibility and validity of ex-vivo 7T MR for the novel application of evaluation of resection margins in TSCC specimens. The results of our study suggest that ex-vivo 7T probably will have a low negative predictive value but a high positive predictive value, meaning that in tumors thicker than a few millimeters we expect to predict whether the resection margin is too small. We believe that this technique is close to practical application, but a randomized controlled trial needs to be performed to confirm our hypothesis that the technique will provide better oncologic and functional outcomes, better quality of life, and lower costs.
